# Current and Emerging Approaches in the Management of Severe Ocular Surface Disease

**DOI:** 10.3390/medicina61101819

**Published:** 2025-10-11

**Authors:** Sandeep K. Dhallu, Molly J. Pritchard, David Y. S. Chau, Stewart B. Kirton

**Affiliations:** 1 Clinical Pharmaceutical and Biological Science, University of Hertfordshire, Hatfield AL10 9AB, UK; 2 Division of Biomaterials and Tissue Engineering, UCL Eastman Dental Institute, Royal Free Campus, Rowland Hill Street, London NW3 2PF, UK; molly.pritchard.21@alumni.ucl.ac.uk (M.J.P.);

**Keywords:** ophthalmology, eye, regenerative medicine, biomaterials, eggshell membrane, amnion

## Abstract

Ocular surface disorders such as dry eye disease are an increasingly encountered ophthalmic disorder, in which signs and symptoms can vary significantly from one patient to the next. Severe dry eye can be a challenge for the ophthalmic practitioner to manage. Contemporary management options are wide-ranging and include topical treatments, contact lenses, and surgical options. More recently, newer stem cell-based therapies have emerged, and early reports have shown promising outcomes. Meanwhile, other novel approaches, such as the eggshell membrane, are currently in development, and while no studies have yet reported on its use in ophthalmic applications, further developments in this area are expected. However, longer-term studies are needed in order to fully assess the safety and efficacy of these newer treatments. There are an increasing number of treatment options available for ocular surface disorders. This article provides an overview of some of the current treatment options that are available for severe ocular surface disorders, including dry eye disease, as well as insight into applications that are currently in development, which may show potential in the future.

## 1. Introduction

Disorders that affect the cornea, limbus, conjunctiva, eyelids, eyelashes, lacrimal apparatus, or tear film are considered to be ocular surface diseases. These can encompass a wide variety of conditions ranging from those that are mild to others that are classed as severe. For instance, Stevens–Johnson syndrome can affect the whole ocular surface, including the corneal and conjunctival epithelial stem cells [1]. Dry eye disease [DED] is a multifactorial disease of the ocular surface [2], whose signs and symptoms can vary greatly across a spectrum, and studies suggest that its prevalence is growing [3].

DED may arise for many different reasons, including underlying inflammation, a deficiency, as a side effect of medication, or from meibomian gland dysfunction (MGD), as well as others. Some biological anti-cancer treatments have been linked to ocular side effects, with the most common being those affecting the ocular anterior segment [4,5,6]. Chemical injury can cause serious damage to the eye, including sight loss, with young males aged 16–25 years old working in industry accounting for the bulk of reported cases [7]. Chemical burns can also cause dry eye as well as keratinisation of the ocular surface, and in specific situations such as this, the DED requires treatment first; otherwise, ocular surface repair options may not provide a sustained benefit [8].

There are many different therapeutic strategies for the treatment of dry eye, and the most appropriate management depends upon its severity. As we gain a deeper understanding of the multifactorial mechanisms that lead to its development, there is a greater focus on treating the underlying source of the disorder. This article will summarise some of the most commonly used management approaches for the treatment of more severe forms of DED and ocular surface disorders. Furthermore, new and emerging areas of research that show promise in providing a safe and effective treatment option for severe ocular surface disorders will be discussed.

## 2. Current and Emerging Management Strategies

### 2.1. Topical

Topical treatments such as lubricating eye drops are usually the first line of treatment, especially in mild-to-moderate cases of dry eye. In more severe cases, topically applied immunosuppressive agents like corticosteroids and immunomodulatory agents such as cyclosporin have been shown to help improve the corneal epithelium, possibly as a result of a decrease in inflammation. Topical cyclosporin A may increase tear production as well as goblet cell density [9]. However, corticosteroid use may be limited due to the associated side effects of raised intraocular pressure, cataracts, and irritation [10,11,12,13].

Autologous serum drops, which are derived from a patient’s own blood, have been developed in order to treat more severe forms of dry eye; however, these come with both benefits as well as limitations. They contain growth factors that promote epithelial healing [14], which lubricating eye drops do not contain, and so have been suggested as being more beneficial for severe forms of DED. However, they are more challenging to prepare, with no universally agreed method for this, and difficult to dispense and store, with contamination risk being an additional factor contributing to their lack of widespread adoption by clinicians [13], as well as being a more costly option for the patient [15]. Blood-derived products have been used successfully for Sjögren's syndrome for 40 years [16]; however, due to the presence of pro-inflammatory factors, the use of allogeneic serum may be a better alternative [17]; however, larger-scale studies are required to investigate their utility further.

The use of whole blood for DED involves the patient undergoing a finger prick to extract a small drop of blood which is then used as a treatment, and when used alongside conventional dry eye therapy was found to improve OSDI scores with no reported adverse effects [18] and improved corneal staining, TBUT, as well as ocular comfort scores [19]. It is a cost-effective and readily available technique, although drawbacks may include patient unwillingness to perform a finger prick multiple times a day [13].

### 2.2. Plasma Rich in Growth Factors [PRGF]

Natural tears have a complex composition and are made up of water, salts, protein and glycoproteins, minor hydrocarbons, lipids, as well as regulatory enzymes and nutrients, proteins, and antimicrobial agents. Eye drops that have been derived from a patient’s own blood have been shown to be effective in treating many ocular surface disorders, having first been applied on the eye nearly fifty years ago [16,20]. Autologous serum [AS] drops were the first blood-derived product used but came with many drawbacks, as detailed above. Plasma rich in growth factors [PRGF] is a platelet-rich plasma with many biological and antimicrobial properties. Some have found PRGF to be more effective than AS in the treatment of moderate-to-severe ocular surface diseases, including dry eye [21,22,23], while others have found no significant difference in outcome measures like OSDI scores, TBUT, and ocular surface staining [24].

### 2.3. Punctal Plugs

Punctal plugs were introduced fifty years ago [25] and work by occluding the punctum, thereby reducing tear drainage in order to retain more moisture at the ocular surface. They are inserted into the punctal ducts in a simple and safe procedure that is reversible [26]. They are recommended for those with more severe forms of dry eye disease, for whom lubricating eye drops are no longer providing effective relief. They have been found to improve symptoms and reduce the use of artificial tears as well as improve the health of the ocular surface. They are a cost-effective treatment option [27] and can be combined with other topical treatments [28]. They are contraindicated in patients with an allergy to the plug material, ectropion, and active ocular infection. Any inflammation, such as that of the lids in blepharitis, should be treated first [14], as the use of punctal plugs on an inflamed ocular surface may lead to toxic tear syndrome [29].

Potential complications include epiphora, foreign body sensation, and local pain/irritation, but this has generally been found to be minimal [26,30]. The development of new materials and designs has helped to improve patient tolerance to punctal plugs, as well as increase their efficacy and reduce the rate of complications [29]. Other issues include potential loss via migration into the lacrimal passageway, although this is uncommon [29,31,32]. Punctal cauterization is an effective option for those who repeatedly lose punctal plugs and can be performed relatively easily in a clinical setting without major complications [33].

### 2.4. Therapeutic Contact Lenses

Contact lenses can play an important role in the management of severe DED as well as in the protection and maintenance of the ocular surface. Soft contact lenses are most commonly used to correct refractive errors; however, they can also be used therapeutically. For example, they may be used to help with the healing of the cornea following injury, burn, or surgery or they may be used to protect the ocular surface from damage in conditions such as trichiasis. Silicone hydrogel lenses can help promote healing following chemical injury [34]. Bandage contact lenses have also been used following cataract surgery to help with wound healing and to improve patient comfort; they have been found to decrease dry eye symptoms and improve OSDI scores [35,36]. A further benefit of bandage contact lenses is in maintaining corneal hydration following keratoprosthesis by stabilising the tear film [37,38]. Contact lenses have also been found to be effective in sealing corneal perforations [39,40,41,42].

With advances in contact lens technology, they can be used in novel ways, such as in drug delivery, and animal studies have found that drug-eluting contact lenses can increase bioavailability by up to 50%, compared to eye drops [43,44]. Furthermore, contact lens sensing technology may be useful in helping to detect ocular biomarkers for DED development [45]. While they show promise as a treatment option for ocular surface disorders, longer-term studies are needed to evaluate their ocular toxicity and biocompatibility in humans.

Additionally, contact lens care and hygiene is of paramount importance to minimise the risk of complications, especially in cases where extended wear is prescribed, and wearers must be educated on how to insert and remove their lenses properly, as well as how to look after them correctly.

### 2.5. Heat, Light, and Low-Level Light Therapy

Warming the eyelids and then massaging the meibomian glands can help manage dry eye symptoms that occur as a result of MGD. This can be performed manually or with automated thermodynamic treatment devices, which have been shown to be effective in managing MGD [46,47].

Light therapies have more recently been developed to treat DED [48]. Intense Pulsed Light [IPL] was first used to treat dermatological conditions such as acne before being used for ophthalmological conditions around 20 years ago [14]. Flashes of light of different wavelengths are applied to the eyelid and surrounding tissues and are absorbed, thereby generating heat. It has been reported that IPL can increase tear film stability, lipid layer thickness [49], and tear break-up times [50]. The exact mechanism by which IPL provides relief is unclear, with several proposed hypotheses, which include the reduction in bacterial load on the eyelids, the eradication of demodex, thrombosis of abnormal blood vessels, the softening of meibum, and the clearing of meibomian glands [51,52,53,54]. Studies suggest that in those with moderate-to-severe symptoms, IPL in combination with meibomian gland expression could be an effective approach for treating DED caused by meibomian gland dysfunction [55,56]. However, it is not recommended in those with dark or deeply pigmented skin due to the risk of skin damage, and other adverse effects include loss of or thinning of eyelashes and eyebrows, corneal epithelial defects, conjunctival irritation, and corneal complications [54]. Exclusion criteria for IPL include those with skin disorders [57] and a history of uveitis and/or herpes simplex virus infection [58]. In general, dry eye management would only be considered once any active infection or inflammation has been resolved.

Low-level light therapy using wavelengths in the visible and near infra-red spectral range has recently been used for MGD, and early studies have shown an improvement in outcomes such as corneal staining [59], as well as other measures like lissamine green staining, the Schirmer test, meibography scores, and a range of tear parameters [54]. Low-level light therapy can be used in conjunction with IPL for MGD, with mixed findings. Some have reported finding improvements in symptoms after combination therapy [60], while others found a greater benefit with IPL but no strong benefit of low-level light therapy [61]. Therefore, further comparative studies are warranted.

### 2.6. Surgical and Transplantation

With more severe forms of ocular surface disease and dry eye conditions, non-invasive treatments are less effective, and surgical intervention is required in order to lower the risk of complications developing, which may affect sight. Depending on the cause, surgical approaches that may be considered include lid-based surgeries such as tarsorrhaphy, upper or lower lid blepharoplasty, levator resection, injection with Botulinum toxin A, conjunctival flap surgery, gland transplantation, as well as others [62]. For example, conjunctivochalasis is a condition that is often misdiagnosed as DED and is characterised by loose conjunctival folds, which can affect tear film stability, leading to dry eye symptoms. Risk factors include age, inflammation, and mechanical friction, and may be more favourably managed surgically as opposed to with other first-line, non-invasive treatment methods such as eye drops, ointments, and bandage contact lenses. Surgical options include conjunctival resection, cauterization, as well as radiowave electrosurgery [13,63].

Tarsorrhaphy is the closure of the eyelids either temporarily or permanently and may be warranted in cases such as persistent epithelial defects in order to help retain the tear film to prevent desiccation. This option has been found to have a quicker healing time than alternatives like amniotic membrane transplantation [64].

Lower lid blepharoplasty is indicated for ectropion and lower lid laxity [65]. Upper lid blepharoplasty can alleviate symptoms of dry eye in the long term without impacting tear film dynamics [66]. However, somewhat counter-intuitively, complications of lid surgery can include an increase in the signs and symptoms of dry eye [67].

Conjunctival disorders, such as pterygium, pinguecula, Stevens–Johnson syndrome, amongst others, may cause DED. The aim of conjunctival flap surgery is the restoration of corneal surface integrity in severe ocular surface diseases. It involves using a thin conjunctival flap to repair the cornea; however, since the evolution of more effective therapeutic options, its use has declined markedly, especially in developed countries [68]. Excision may be indicated for pterygium and pinguecula, followed by conjunctival or amniotic membrane grafting [62].

The lacrimal and salivary glands have a similar structural composition and autonomic innervation [69]. Therefore, transplantation of glands like the salivary glands or the secretory duct of the parotid duct gland may be an option to restore lubrication to the ocular surface in certain, more severe cases where other treatment options have failed. However, there may be complications associated with parotid gland transplantation surgery, and so this is usually reserved as an endpoint treatment option [62]. Potential adverse effects of parotid gland transplantation include atrophic and degenerative changes in the transplanted glands, infection, fistula formation, duct obstruction, amongst others [13]. Transplantation of minor salivary glands into the upper or lower conjunctival fornix is simpler in comparison and can improve outcome measures such as ocular surface staining, TBUT, and visual acuity, with reported postoperative complications including graft necrosis, ptosis, and donor site granuloma formation [70].

### 2.7. Gene Therapy

This technique refers to the transfer of genetic material in order to treat disease [71]. While the concept has existed for nearly half a century, it has more recently gained traction as a treatment option for various human diseases, including those affecting the anterior eye. Applications include conditions that cause abnormal wound healing, inflammation, corneal cloudiness, scarring, and graft rejection [72]. Many autoimmune diseases like Sjögren’s syndrome, rheumatoid arthritis, and lupus are associated with DED, and animal studies have shown promising results of gene therapy in restoring tear production and reducing corneal surface defects [73]. Further trials translating gene therapy into human clinical trials are needed.

### 2.8. Cell-Based Therapies

Persistent corneal epithelial defects may occur for a multitude of different reasons, including severe dry eye, and these corneal defects may lead to corneal opacification and loss of vision. Cell-based therapies using both corneal and non-corneal stem cells have been used to replace damaged corneal epithelial cells and regenerate the ocular surface [74,75,76].

**Figure 1:**
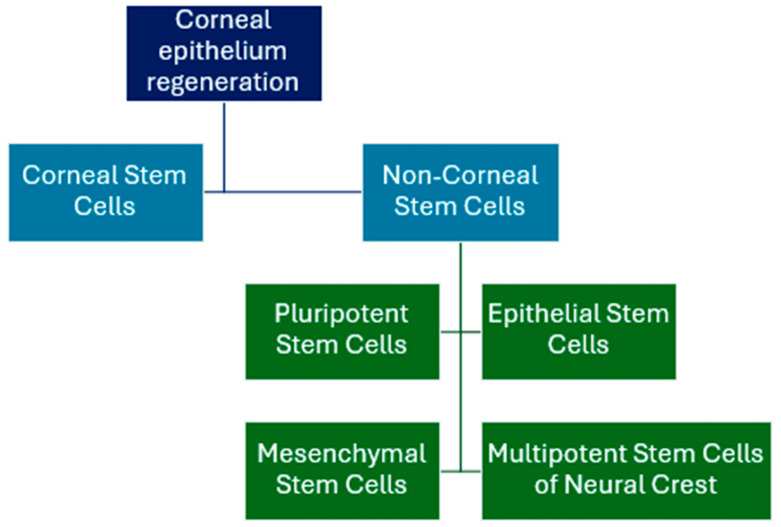
Corneal and non-corneal stem cells used in corneal epithelium regeneration [76].

Different types of stem cells have been used for corneal surface regeneration [77], including both corneal and non-corneal, which can be sub-classified into four groups [Figure 1]. Non-corneal stem cells are able to differentiate into cells that have characteristics of corneal epithelial cells. Pluripotent cells are derived from embryonic stem cells, epithelial stem cells from oral mucus, amniotic membrane, epidermis, and hair follicles, mesenchymal stem cells from bone marrow, adipose tissue, amniotic membrane, placenta, and umbilical cord, and neural crest origin stem cells from dental pulp stem cells [76].

### 2.9. Amniotic Membrane and Amniotic Drops

There are various sources of corneal and non-corneal stem cells. Foetal and oral cavity tissues are examples of tissues that are abundant in stem cells, and foetal tissue in particular has gained interest in regenerative therapies for ocular surface disorders. The placenta is normally treated as waste material and discarded after birth; however, amnion, which is the innermost epithelial layer of the placenta, can be harvested and used as transplant material [78]. The amniotic membrane can be obtained after elective caesarean deliveries and processed into a graftable material [79]; however, it requires specialised storage and application.

Amniotic membrane transplantation [AMT] was first used surgically in 1910 as a substitute material for a skin graft. It was first used for ocular applications in the 1940s and then largely abandoned until the 1990s when it regained popularity amongst ophthalmologists [80,81]. It has anti-inflammatory properties [82,83,84], antibacterial effects against a range of bacteria, as well as anti-viral properties [85,86,87,88]. AMT has been used in the treatment of corneal ulcers, certain glaucoma cases, after excision of malignant and benign tumours, in oculoplastics, for pterygium, as well as in strabismus surgery. It has also been used in the treatment of chemical burns, Steven–Johnson syndrome, graft versus host disease, and recurrent corneal erosion, and shows promise as a treatment option for advanced DED [14,78,89,90].

The cryopreserved amniotic membrane has been shown to improve the ocular surface recovery in those with moderate-to-severe DED after 1 month [90] and 3 months [91]. A novel specialised bandage contact lens, which uses a room-temperature stable dehydrated amniotic membrane that is applied using a specialised bandage contact lens, has been developed, which enables suture-less application, and early research has shown promise for the treatment of moderate-to-severe DED, with an improvement in symptoms and a decrease in ocular surface signs of stress after 1 month [90]. Dehydrated amniotic membrane can also be applied with standard soft disposable contact lenses, and similar results have been found demonstrating favourable epithelial recovery in those with severe DED [92]. Sutureless applications of the amniotic membrane are gaining popularity for both DED [91,93] and other disorders, including ulcerative keratitis, persistent corneal epithelial defects, chemical and thermal burns, neuropathic corneal pain, and others [94,95,96,97,98,99]. It may offer economic benefits to clinical services compared to some current standard treatment options, such as cyclosporin, for severe DED, as well as better reported clinical outcomes [100].

AMT has been reported to provide sustained symptomatic improvement in dry eye patients with reduced corneal and conjunctival staining, and some report improved corneal nerve density [91,96,101,102,103,104].

A novel approach that extracts amniotic membranes to be re-hydrated so that they can be applied topically as drops has been developed, and early results show promise in their safe and effective use for reducing symptoms and clinical signs of severe ocular surface disease, persistent epithelial defects, and corneal ulcers [105,106,107,108]. However, further studies are needed.

### 2.10. Egg Shell Membrane

There are similarities between skin and cornea wound healing. The treatment for chronic skin wounds often calls for regular application of topical drugs, and this is also often the case with chronic ocular surface disorders. Adherence can be problematic, and thus, the development of a two-in-one ocular bandage could prove useful to a large proportion of the population, particularly older people or those who struggle with administering drops due to dexterity or for other reasons. A drug-incorporated bandage could also be useful for the treatment of chronic wounds to relieve pain, protect the ocular surface, promote corneal healing and epithelial regeneration, and deliver ophthalmic drugs on the ocular surface. However, such a bandage would need to consider the physical and surface properties such as thickness, transparency, modulus, wettability, water content, oxygen permeability, and maximal drug loading capacity [109].

**Figure 2:**
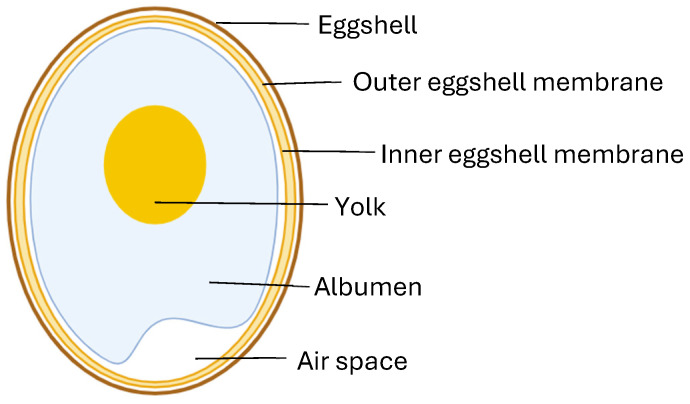
Schematic outlining the structure of the eggshell membrane in relation to a typical egg.

The eggshell membrane [ESM] is a naturally occurring material from the poultry and food industries, which has recently gained attention for its use in biomedical and healthcare applications due to its unique physical and biological properties. In essence, the ESM is a clear film that lines the eggshell [ES] and consists of the inner side membrane, also termed the limiting membrane, and the outer membrane [Figure 2].

As a whole, the ESM has a significantly high protein load of approximately 80–85%, where at least 500 different types of proteins have been identified, of which the collagen superfamily provides the major structural component [110]. Crucially, the outer ESM contains only collagen type I, whereas the inner ESM contains both collagen type I and V, thus dictating the translational applications of the substrate [111,112]. Moreover, the ESM procures a wide range of bioactive components, such as fibronectin, proteoglycans, and glycoproteins, as well as other inorganic components, during its formation and ageing process, as summarised in Table 1.

The ESM closely resembles the structure and composition of the extracellular matrix [ECM], exhibits semi-permeability, porosity, is non-toxic and biodegradable, has anti-inflammatory and antibacterial properties, and so naturally lends itself towards biomedical translational applications [109]. It benefits from low costs, material availability, and accessibility, as well as reduced ethical concerns. Recently, microparticles were incorporated into ESM to produce a cheap, effective, and rapid wound bandage for patients. The generated bandage was characterised by evaluating the physical, mechanical, and biological properties and reported to show good biocompatibility and did not promote pro-angiogenesis. Although the bandage exhibited promising cornea wound healing properties, further work investigating in vivo wound healing will need to be undertaken to validate its effectiveness [120].

Although it may seem that the ESM is presented as a novel material in medical research, historical biomedical uses of the ESM have been reported; for example, within traditional Chinese medicine, the ESM was used to treat burns, ulcers, and tympanic perforations. In ophthalmology, the first use of the ESM is believed to date back to 1899 for the treatment of symblepharon, ocular burn, corneal ulcer, and iritis. Here, raw ESM was applied directly to the wounds, with the resulting outcomes being reduced irritation, pain, and infection risk, as well as improved healing and recovery [121]. In hindsight, this study can be considered to be a pioneer in paving the first steps for the use of ESM as a scaffold to facilitate the growth and repair of ocular tissues. Recently, Choi and colleagues [122] used the ESM to regenerate the retinal pigment epithelium by incorporating it within gellan gum. Mensah and coworkers further explored the ESM as a potential material for corneal wound healing due to its innate characteristics of transparency, porosity, and permeability, with a study demonstrating that the ESM is capable of successfully supporting the attachment and proliferation of immortalised corneal epithelial cells and corneal mesenchymal stromal cells [120]. A number of complementary studies by the same team also considered incorporating drug and/or growth factor-loaded microparticles, as well as embedding silver nanoparticles within the ESM to further increase the therapeutic efficacy or enhance the antimicrobial capabilities of the membrane, respectively [123,124,125].

While no other studies have yet reported on the use of ESM in ophthalmic surgery or other ocular applications, current research is promising, and it seems that the future direction of ESM-based research includes its enhancement to improve the desired function required for its ultimate endpoint application, and as such, further developments in this area are expected.

## 3. Conclusions

The ocular surface is a complex structure that may be altered by disease, giving rise to painful ocular signs and symptoms in the patient. There are many different therapeutic strategies for the treatment of severe dry eye, and this is currently an area of active research, with many new and emerging treatment options in development. Recently, regeneration therapies have emerged as a promising approach to repairing the damaged ocular surface. In advanced cases with severe DED, where conventional therapies may not provide effective relief, more surgical intervention may be required. Management options should consider the underlying contributing causes and should consider the severity of the disease in order to provide safe, lasting, and effective relief.

## Figures and Tables

**Table 1:** Biomolecules found within the ESM that allow translation of the ESM into ocular-specific tissue engineering applications.

ESM Component		Benefits of Molecules in Tissue Engineering	References
Collagen		- Acts as a scaffold to facilitate cell seeding and adherence - Type V collagen maintains structural integrity and tensile strength of the ESM	[113,114]
Fibronectin		- Facilitates cellular adhesion, growth, migration, and repair	[115]
Osteopontin		- Has an immunomodulatory effect on the host by regulating cytokine release and macrophage recruitment	[116,117]
Glycosaminoglycans	Hyaluronic acid	- Maintains viscoelasticity via water retention, promotes ECM secretion, reduces inflammation, and is involved in every step of wound healing	[118]
	Heparin	- Has a high affinity to growth factors, which allows easy modification of ESM to promote growth and healing	
	Chondroitin sulfate	- Promotes cellular adhesion and induces cellular differentiation	
	Keratan sulfate	- Aids the control of charge and ion gradients needed for cellular adhesion, proliferation, and differentiation	
Amino Acids		- Amino acids found in high abundance in the ESM include proline, cysteine, glycine, glutamine, and asparagine - Important for collagen and protein synthesis needed to maintain the ECM matrix - Impacts metabolic and physiologic processes such as cellular proliferation which can act as an energy substrate	[119]

## Data Availability

No new data were created or analyzed in this study.
